# Involvement of Noncoding RNAs in the Differentiation of Osteoclasts

**DOI:** 10.1155/2020/4813140

**Published:** 2020-08-25

**Authors:** Yi Zhao, Lingfei Jia, Yunfei Zheng, Weiran Li

**Affiliations:** ^1^Department of Orthodontics, Peking University School and Hospital of Stomatology, Beijing 100081, China; ^2^Department of Oral and Maxillofacial Surgery, Peking University School and Hospital of Stomatology, Beijing 100081, China; ^3^Central Laboratory, Peking University School and Hospital of Stomatology, Beijing 100081, China

## Abstract

As the most important bone-resorbing cells, osteoclasts play fundamental roles in bone remodeling and skeletal health. Much effort has been focused on identifying the regulators of osteoclast metabolism. Noncoding RNAs (ncRNAs) reportedly regulate osteoclast formation, differentiation, survival, and bone-resorbing activity to participate in bone physiology and pathology. The present review intends to provide a general framework for how ncRNAs and their targets regulate osteoclast differentiation and the important events of osteoclastogenesis they are involved in, including osteoclast precursor generation, early differentiation, mononuclear osteoclast fusion, and multinucleated osteoclast function and survival. This framework is beneficial for understanding bone biology and for identifying the potential biomarkers or therapeutic targets of bone diseases. The review also summarizes the results of *in vivo* experiments and classic experiment methods for osteoclast-related researches.

## 1. Introduction

### 1.1. A Brief Introduction to Osteoclasts

Multinucleated osteoclasts are closely related to orthodontic tooth movement, as they can resorb mineralized bone and, together with osteoblasts, initiate the bone remodeling cycle [1]. Osteoclasts are also essential for continuous tooth movement as they remove adjacent bone and the necrotic tissue generated by periodontal ligament hyalinization [2, 3]. Moreover, bone is remodeled throughout life, which consists of osteoclasts resorbing old bone and osteoblasts synthesizing new bone. When the balance between these two processes is broken, many skeletal disorders occur [4, 5]. Enhanced osteoclast formation and activity contribute to bone loss of osteoporosis, rheumatoid arthritis, and osteolysis of metastatic tumors [6–8].

**Figure 1:**
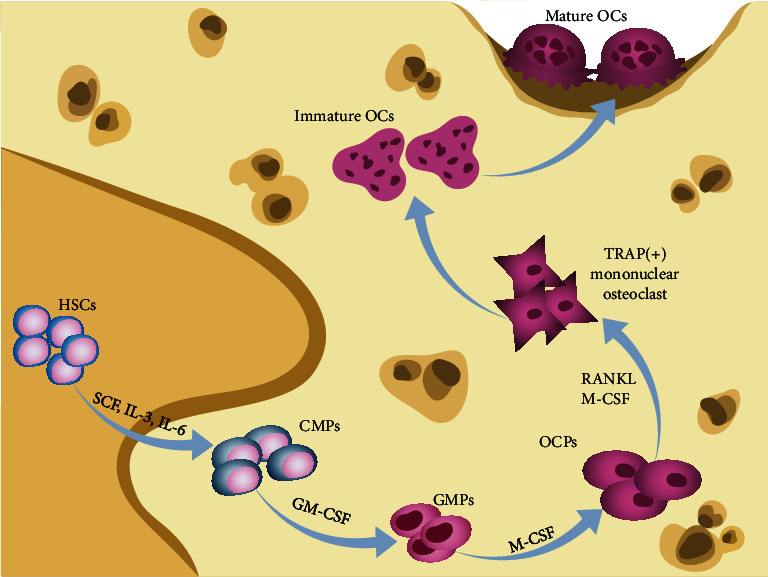
Osteoclastogenesis. Within the bone marrow, SCF, IL-3, and IL-6 stimulate HSCs to differentiate into CMPs. GM-CSF stimulates CMPs to differentiate into GMPs. M-CSF stimulates GMPs to differentiate into OCPs. OCPs express RANK and CSF1R. Upon RANKL and M-CSF binding to RANK and CSF1R, respectively, OCPs gradually differentiate to TRAP-positive mononuclear osteoclasts which then fuse to become multinucleated osteoclasts. Immature osteoclasts then undergo morphological changes and become mature and activated osteoclasts.

Osteoclasts are derived from macrophages and monocytes of hematopoietic lineage [5]. Within the bone marrow, stimulation of hematopoietic stem cells (HSCs) by stem cell factor (SCF), interleukin- (IL-) 3, and IL-6 yields common myeloid progenitors (CMPs). Then, stimulated with granulocyte/macrophage colony-stimulating factor (GM-CSF), CMPs differentiate into granulocyte/macrophage progenitors (GMPs). After being stimulated with macrophage colony-stimulating factor (M-CSF), GMPs further differentiate into cells of monocyte/macrophage lineage, which are considered osteoclast precursors (OCPs). OCPs express receptor activator of nuclear factor *κ*B (RANK) and colony-stimulating factor 1 receptor (CSF1R) [9]. Upon receptor activator of nuclear factor *κ*B ligand (RANKL) and M-CSF binding to RANK and CSF1R, respectively, OCPs then further differentiate into TRAP-positive mononuclear osteoclasts, which subsequently fuse to become multinucleated osteoclasts [10]. The sequential expression of c-Fos, nuclear factor *κ*B (NF-*κ*B), and nuclear factor of activated T cell cytoplasmic 1 (NFATc1), tartrate-resistant acid phosphatase (TRAP), cathepsin K (CTSK), integrin *β*3 (ITGB3), and calcitonin receptor (CALCR) are induced by RANKL and M-CSF [5]. They play important roles during these processes and lead to the formation of mature and activated osteoclasts, which marks the completion of osteoclastogenesis [11] (Figure 1).

### 1.2. A Brief Introduction to Noncoding RNAs

Noncoding RNAs (ncRNAs) are defined as RNAs that do not encode proteins [12] and include “housekeeping” rRNAs, tRNAs, small nuclear RNAs, and small nucleolar RNAs (snoRNAs) [13]. But ncRNAs are far more than these. Studies have shown that there are only approximately 20,000 protein-coding genes in the human genome, while at least 90% of the genome is actively transcribed which implies that about 80% of the genome is functional noncoding genes [14]. Although the transcripts of some such genes are suspected to be transcriptional noise, ncRNAs are important biological determinants and are recognized as a breakthrough for disease treatment in mechanism research, drug development, and new biomaterial invention [13, 15]. In physiology and development of life, they control gene expression in multiple levels, including chromatin architecture/epigenetic memory, transcription, RNA splicing, editing, translation, and turnover [12]. ncRNAs are subdivided into two major classes according to their size: small ncRNAs (<200 nt) and long ncRNAs (lncRNAs, >200 nt) [14]. Small ncRNAs consist of microRNAs (miRNAs) which regulate posttranslational gene silencing, piwi-interacting RNAs (piRNAs) which silence retrotransposable elements in the genome and regulate DNA methylation, snoRNAs which modify RNAs other than rRNAs, etc. [16–18]. lncRNAs, comprising long or large intergenic ncRNAs (lincRNAs), circular RNAs (circRNAs), etc., are involved in chromatin remodeling, transcriptional regulation, and posttranscriptional processing and thus play important roles in regulating gene expression during development, differentiation, and disease [16, 19, 20].

Since miRNA is the most widely studied type of ncRNAs, its naming is more orderly. miRNA names include sequential numerical identifiers to distinguish them from each other [21]. Lettered suffixes indicate that the miRNAs differ at only one or two positions in paralogous sequences—for instance, miR-29a, miR-29b, and miR-29c [21]. And “5p” or “3p” at the end of miRNA names indicate that they are processed from the 5′ and 3′ end arms—for example, miR-142-5p and miR-142-3p [21].

## 2. Literature Collection

The research was carried out in the MEDLINE database (PubMed research engine): ((((miRNA) OR lncRNA) OR ncRNA) AND ((osteoclast differentiation [tw]) OR (osteoclastogenesis))). This search retrieved 1577 articles. Then, considering that most of the ncRNA researches were carried out after 2008, we narrowed the publish date to January 1, 2008, to March 1, 2020. We also excluded reviews and added additional filter criteria of language, using the search strategy as follows: ((((((miRNA) OR lncRNA) OR ncRNA) AND ((osteoclast differentiation [tw]) OR osteoclastogenesis))) NOT “review”[Publication Type]) AND (“2008/01/01”[PDat]: “2020/03/01”[PDat]) AND English [lang]). This search retrieved 1352 articles. Afterwards, we manually assessed the titles and abstracts of the collected articles and excluded those not relevant, which means that the articles are not mainly focused on the relationship between ncRNAs and osteoclast differentiation or could not present enough or clear evidence to prove the relationship between them. Finally, we manually added some classical literature and essential articles, which would be helpful for explaining ncRNAs' regulatory roles in osteoclastogenesis.

## 3. Involvement of ncRNAs in Osteoclast Differentiation

The factors that regulate osteoclastogenesis, such as ncRNAs, natural compounds, and epigenetic mechanisms, have been investigated [22]. *In vitro* experiment showed that the expression levels of 518 lncRNAs, 207 mRNAs, 37 miRNAs, and 24 circRNAs fluctuated during osteoclastogenesis of murine RAW264.7 cells [22], which indicated a role of ncRNAs in the regulation of osteoclastogenesis. For example, osteoclasts cannot be produced from bone marrow HSCs deficient in mature miRNAs [23–26]. Moreover, small interfering RNA- (siRNA-) mediated silencing of the Drosha enzyme complex (which promotes miRNA biosynthesis) or the RNA-induced silencing complex (RISC) (which mediates miRNA binding to its target messenger) blocks osteoclastogenesis [23, 27].

Table 1 summarizes a list of osteoclast-related ncRNAs with validated targets and functions in regulating osteoclastogenesis.  Table 2 summarizes a list of ncRNAs implicated in biological events in osteoclasts in *in vivo* studies.

**Figure 2:**
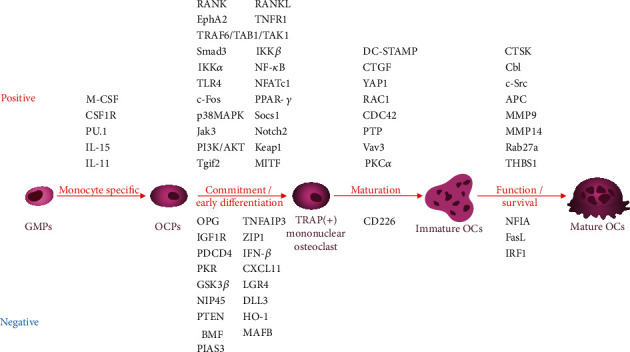
Important regulators involved in ncRNAs regulating osteoclastogenesis. ncRNAs function through various regulators to participate in different stages of osteoclastogenesis, including OCP generation, early differentiation, mononuclear osteoclast fusion, and multinucleated osteoclast function and survival. The regulators are positioned by the stage of osteoclastogenesis they are mainly involved in. Positive regulators are listed above the cell figures, while negative regulators are listed below them.

In the following sections, we will discuss miRNAs and lncRNAs according to their functioning stages in osteoclastogenesis (Figure 2). To our knowledge, ncRNAs and their targets are usually involved in more than one stage of osteoclastogenesis. To give a more accurate network of ncRNAs, we tend to classify the functioning stages of ncRNAs according to their mostly described functions.

### 3.1. Involvement of ncRNAs in OCP Generation

Osteoclasts are derived from HSCs, which give rise to CMPs, and the latter yield GMPs. GMPs further differentiate into macrophage/osteoclast/dendritic cell precursors, which are known as OCPs [9].

#### 3.1.1. M-CSF, CSF1R, and PU.1

The differentiation from early-stage CSF1R^–^ osteoclast progenitors to late-stage CSF1R^+^ OCPs involves various differentiation, proliferation, and antiapoptotic signals. These signals are initiated by M-CSF produced by the neighboring cells of osteoclasts, including osteoblast and bone marrow stromal cells. M-CSF could induce the proliferation of OCPs, promote cell survival, and upregulate the expression of RANK [28]. In this process, circRNA_28313, miR-195a, and M-CSF together form a circRNA-miRNA-mRNA axis, in which circRNA_28313 relieves miR-195a-mediated suppression on M-CSF by acting as a competing endogenous RNA, therefore modulating osteoclastogenesis of bone marrow-derived macrophages (BMMs) [29].

Besides M-CSF, CSF1R and PU.1 also play key roles in the generation of OCPs. CSF1R could transmit M-CSF signals. And PU.1, which is a member of the Ets family, could regulate CSF1R transcription through Ets-binding sites on a CSF1R promoter. It was observed that deletion of CSF1R and PU.1 resulted in the lack of both macrophages and osteoclasts [30]. Overexpression of miR-155 suppresses PU.1 expression and activity, resulting in inhibited TRAP expression, osteoclast differentiation, and bone-resorbing activity, as well as repressed mononuclear osteoclast fusion and syncytia formation [31].

#### 3.1.2. IL-15

IL-15 stimulates the differentiation of osteoclast progenitors into OCPs. The possible mechanism might be that IL-15 induces RANKL expression in osteoblasts and stromal cells, indirectly promoting osteoclastogenesis. Moreover, IL-15 is reported to exhibit a synergistic effect with RANKL in osteoclast differentiation by inducing activation of extracellular signal-regulated kinase (ERK), a specific subtype of mitogen-activated protein kinase (MAPK) [32]. As miR-212 negatively targets IL-15, the inhibition of miR-212 gives rise to upregulated IL-15 and thus promotes osteoclastogenesis [33].

#### 3.1.3. IL-11

IL-11 is identified as a necessary factor that promotes osteoclast differentiation. The possible mechanism involves the downstream JAK1/signal transducer and activator of transcription-3 (STAT3) signaling pathway, in which activated STAT3 induces the expression of c-Myc, an important factor in osteoclastogenesis [34]. IL-11 also downregulates GM-CSF expression and sustains the pool of osteoclast progenitors [32, 35]. During the process of osteoclastogenesis, GM-CSF reveals duration-relevant biphasic effects as it exhibits positive effect on osteoclast proliferation and formation in short-term treatment while it inhibits osteoclastogenesis in long-term treatment [36]. So IL-11 might work as an inhibitor of GM-CSF long-term effect and thus promotes osteoclast differentiation. miR-124 plays a negative role in cancer-induced osteoclast differentiation and activity through targeting IL-11 [37].

### 3.2. Involvement of ncRNAs in the Early Differentiation of OCPs

During this stage of osteoclastogenesis, the change of OCPs is mainly manifested in the interaction and expression of internal molecules, and more osteoclast-specific molecules are expressed.

#### 3.2.1. RANK/RANKL and Related Molecules

RANK and CSF1R are both transmembrane signaling receptors required for osteoclast differentiation and activation. RANKL and M-CSF bind to and activate RANK and CSF1R, respectively, which initiates the early stage of osteoclastogenesis [10]. Downregulation of miR-503 and miR-144-3p facilitates this stage of osteoclastogenesis by targeting RANK. Conversely, their overexpression inhibits osteoclastogenesis, demonstrated by reduced TRAP staining, decreased TRAP-positive cell numbers, and suppressed expression of osteoclast-specific genes [38, 39]. Overexpression of lncRNA-NONMMUT037835.2 is also reported to inhibit osteoclastogenesis, as NONMMUT037835.2 could downregulate RANK expression and the subsequent NF-*κ*B/MAPK signaling pathway [40].

Like M-CSF, RANKL is also an exogenous regulatory factor for osteoclastogenesis generated from neighboring cells. RANKL exists as a membrane-bound or secreted protein of osteoblasts and activated T cells [11]. miR-106b and miR-338-3p weaken osteoclastogenesis by negatively targeting RANKL and are involved in RANKL-RANK signaling [41, 42]. However, the positive effects of miR-106b and miR-338-3p are also reported, which may be due to the tissue specificity of osteoclastogenesis [43, 44].

Integrin subunit *α*4 (ITGA4, also known as CD49d), one of the targets of miR-let-7e, is involved in osteoclastogenesis through several indirect ways [33]. Binding of ITGA4 to its receptor increases RANKL production of stromal/osteoprogenitor cells and therefore facilitates osteoclast formation. The interaction with fibronectin is also a possible pathway for ITGA4 to regulate osteoclastogenesis [45, 46]. miR-let-7e negatively targets ITGA4, and inhibited miR-let-7e causes the upregulation of ITGA4. However, inhibited miR-let-7e is observed to suppress osteoclastogenesis, whose mechanism is still unclear [33]. The possible explanation may be the crosseffect of both the RANKL/RANK pathway and fibronectin.

Additionally, there are some other ncRNAs regulating osteoclastogenesis directly or indirectly by the RANKL/RANK pathway. Transducer of ERBB2, 2 (TOB2), a potential target of miR-422a, is also involved in osteoclast differentiation indirectly as it attenuates RANKL expression in stromal cells [47, 48]. The upregulation of miR-145-5p increases the expression of RANK and RANKL and supports RANKL-RANK signaling by directly targeting osteoprotegerin (OPG), which can bind to and neutralize RANKL [49]. miR-100-5p suppresses osteoclast differentiation and decreases the expression of osteoclast-specific gene expression, as well as the protein level of RANK and RANKL, by targeting fibroblast growth factor 21 (FGF21) [50], which is able to alter the RANKL/OPG ratio and promote osteoclastogenesis by indirect effects through other cell types or secreted factors [51].

Ephrin type-A receptor 2 (EphA2) is suggested to be a miR-141 target in rhesus monkey osteoclasts [52]. Excessive expression of EphA2 in OCPs induces osteoclast differentiation. And EphA2 is involved in osteoblast-osteoclast interaction as the ratio of RANKL/OPG decreases in osteoblasts lacking EphA2. There is also some hypothesis that EphA2 may participate in osteoclast autoregulation between osteoclasts at different stages of differentiation [53].

#### 3.2.2. TRAF6/TAB1/TAK1 and Related Molecules

RANKL-RANK interaction induces the formation of a complex composed of RANK, tumor necrosis factor receptor-associated factor 6 (TRAF6), transforming growth factor-*β*-activated kinase 1-binding protein 1 (TAB1), and transforming growth factor-*β*-activated kinase 1 (TAK1). The complex leads to the activation of endogenous TAK1, which stimulates the activation of NF-*κ*B and its downstream NFATc1 subsequently [54].

Downregulation of miR-125a, miR-219, and miR-146a is reported to promote the early stage of osteoclastogenesis by directly targeting TRAF6, whereas NFATc1 inhibits miR-125a transcription by binding to its promotor region and forms a TRAF6/NFATc1/miR-125a regulatory feedback loop [55–57]. TRAF6 degradation is under regulation of both CX3C chemokine receptor 1 (CX3CR1) and tumor necrosis factor-*α*-induced protein 3 (TNFAIP3). Upregulation of miR-212, miR-125, and miR-let-7e elevates TRAF6 expression as miR-212 targets CX3CR1 and miR-125 and miR-let-7e target TNFAIP3 [33].

Additionally, miR-218 attenuates osteoclastogenesis through targeting tumor necrosis factor receptor 1 (TNFR1), which is involved in the activation of TAK1 and phosphorylated p65 [58]. The TRAF6-TAB1-TAK1 complex also recruits mothers against decapentaplegic homolog 3 (Smad3), which is necessary for the complex formation and downstream signal transduction [59]. Downregulation of miR-145, which targets Smad3, augments Smad3 expression and activity. Overexpression of miR-145 suppresses osteoclast differentiation and the expression of markers of osteoclast activity [60].

#### 3.2.3. NF-*κ*B and Related Molecules

NF-*κ*B is a key regulator of osteoclastogenesis, of which the induction is a hallmark event in the cell fate determination of osteoclasts. In the canonical pathway of NF-*κ*B activation, TAK1 phosphorylates and activates inhibitor of NF-*κ*B kinase subunit *β* (IKK*β*). IKK*β* together with IKK*α* then phosphorylates and degrades inhibitor of NF-*κ*B (I*κ*B), resulting in an NF-*κ*B dimer entering the nucleus [61].

Downregulation of miR-338-3p accelerates the activation of NF-*κ*B by targeting IKK*β*, and IKK*α* is reported to be a potential target of miR-223 [62–64]. TNFR2 (encoded by tumor necrosis factor receptor superfamily member 1B, TNFRSF1B) regulates osteoclastogenesis by transducing intracellular signals which leads to the proteolytic breakdown of I*κ*B and the activation of NF-*κ*B [65]. However, by targeting TNFRSF1B, upregulation of miR-125a-5p inhibits the expression of TNFR2 and promotes osteoclast differentiation, growth, migration, and invasion [66].

Upregulation of miR-99b gives rise to enhanced NF-*κ*B activity as it is reported to target insulin-like growth factor 1 receptor (IGF1R), whose signaling impedes NF-*κ*B activity [33, 67]. IGF1, the ligand of IGF1R, is required for normal osteoclast differentiation demonstrated by IGF1 knockout presenting a decreased osteoclast number [68]. IGF1 is also a possible target of miR-422a [47]. IGF2 is a peptide hormone that can modulate osteoclastogenesis through IGF1R activation and may participate in osteoblast-osteoclast interactions [69, 70]. IGF2 is thought to be the likely target of miR-483-5p promoting osteoclast differentiation and inhibiting apoptosis as miR-483-5p directly inhibits the expression of IGF2 and IGF2 could in turn reverse the effects of miR-483-5p [71].

Toll-like receptor 4 (TLR4), together with myeloid differentiation primary response gene 88 (MyD88), ultimately activates NF-*κ*B [72]. miR-218 and miR-618 suppress osteoclastogenesis by targeting TLR4 and restraining the TLR4/MyD88/NF-*κ*B signaling pathway [73]. ZIP1, a plasma membrane zinc transporter, impedes both NF-*κ*B binding activity and osteoclast resorptive activity [74]. Solute carrier family member 1 (SLC39A1), the encoding gene of ZIP1, is a potential target of miR-133a [75]. miR-378 is also involved in the regulation of osteoclastogenesis as both knockdown and overexpression of miR-378 lead to suppressed osteoclast formation. One of the hypothetic molecular mechanisms is that miR-378 is involved in the activation of the NF-*κ*B signaling pathway through regulating the expression of soluble intracellular adhesion molecule in serum [55, 76–78].

#### 3.2.4. NFATc1 and Related Molecules

NFATc1 is an essential transcription factor for osteoclastogenesis, which is inducted mainly through two ways—initial induction and autoamplification. In the initial induction, activated NF-*κ*B is recruited to the NFATc1 promoter and cooperates with NFATc2 to activate the initial induction of NFATc1. The autoamplification refers to RANKL-RANK binding cooperating with immunoreceptors to activate the calcium signals, which induces NFATc1 autoamplification via calcineurin. Activator protein 1 (AP-1, containing c-Fos and c-Jun), which is recruited to the NFATc1 promoter concomitantly, is also critical for this autoamplification [79].

NFATc1 expression and activation are supported by the downregulation of miR-124 as it directly targets NFATc1 [80, 81]. In the meantime, through the inhibition of miR-124, endothelial progenitor cell- (EPC-) derived exosomal lncRNA-MALAT1 induces migration and osteoclastogenesis of BMMs [82]. miR-1897 also targets NFATc1 and downregulates its expression. And lncRNA-MIRG functions as a molecular sponge to modulate the inhibitory effect of miR-1897 on NFATc1 and therefore enhances NFATc1 expression [83].

Downregulation of miR-27a enhances c-Fos activity by targeting peroxisome proliferator-activated receptor *γ* (PPAR*γ*), a direct regulator of c-Fos expression. The enhanced c-Fos activity then induces NFATc1 autoamplification and thus promotes osteoclastogenesis [84, 85]. Upregulation of miR-21 augments NFATc1 activity by targeting programmed cell death 4 (PDCD4), of which the diminution lifts the repression on c-Fos and forms the c-Fos/miR-21/PDCD4 positive feedback loop [23, 26]. Downregulation of miR-218 facilitates NFATc1 activity through the p38MAPK-c-Fos-NFATc1 pathway, as miR-218 inhibits the phosphorylation of p38MAPK and thus downregulates the expression of NFATc1 and c-Fos [32, 86].

Interferon *β* (IFN-*β*) interferes with the expression of NFATc1 by inhibiting the expression of c-Fos [87]. Upregulation of miR-182 increases NFATc1 activation by targeting protein kinase double-stranded RNA-dependent (PKR), which is a positive regulator of IFN-*β* [88]. miR-155 could suppress NFATc1 activation as it targets suppressor of cytokine signaling 1 (Socs1), a negative regulator of IFN-*β* [89–91]. miR-133a, which potentially targets chemokine (C-X-C motif) ligand 11 (CXCL11) and chemokine (C-X-C motif) receptor 3 (CXCR3), has a positive effect on osteoclastogenesis [75, 92]. CXCL11, upregulated by IFN-*β*, suppresses osteoclast differentiation in a dose-dependent manner and is thought to mediate the regulation of osteoclastogenesis by IFN-*α* and IFN-*β* [93]. However, CXCR3, the receptor of CXCL11, lacks direct evidence for being involved in osteoclastogenesis.

Glycogen synthase kinase-3*β* (GSK3-*β*) is reported to downregulate NFATc1 expression and regulate NFATc1 nuclear localization as GSK3-*β* is capable of blocking NFATc1 transcription and regulating RANKL-induced Ca^2+^ oscillation-mediated NFATc1 activation [94]. Phospho-GSK3-*β* is the inactive form of GSK3-*β*. miR-23a attenuates NFATc1 activity by targeting GSK3-*β* and inhibiting its phosphorylation [95]. Leucine-rich repeat-containing G protein-coupled receptor 4 (LGR4), as a competitor of RANK for RANKL, has the ability to inhibit canonical RANK signaling and activate the GSK3-*β* signaling pathway [96]. Upregulation of miR-34c promotes NFATc1 activity by regulating GSK3-*β* signaling via targeting LGR4 [97].

lncRNA-AK077216 promotes the differentiation, fusion, bone resorption, and actin ring formation of osteoclasts while it reduces OCP apoptosis by upregulating NFATc1 through repression of NFAT-interacting protein (NIP45) expression, which is reported to interact with NFATc2 and TRAF6 [98, 99]. lncRNA-Jak3 knockdown inhibits monosodium urate monohydrate- (MSU-) induced osteoclastogenesis and leads to an inhibition of osteoclast bone-resorbing activity, as shown by decreased osteoclast number and pit size. The functional role of lncRNA-Jak3 in osteoclastogenesis is played by Janus kinase 3 (Jak3). lncRNA-Jak3 is coexpressed with Jak3, which positively regulates NFATc1 [100].

lncRNA-LINC00311 has been implicated in osteoclastogenesis by inducing osteoclast proliferation and differentiation while suppressing apoptosis via targeting delta-like 3 (DLL3), whose downregulation activates the Notch signaling pathway. In this way, overexpression of LINC00311 elevates the expression levels of neurogenic locus notch homolog protein 2 (Notch2) and TRAP [101]. Notch2 has been shown to interact with NF-*κ*B, and both of them are recruited to the NFATc1 promoter and enhance the expression of NFATc1 [102, 103]. The knockdown of lncRNA-DANCR could reduce the promotion effect of compression force on osteoclastogenesis via Jagged 1, a ligand of Notch2. DANCR is also reported to promote the expression of IL-6 and tumor necrosis factor-*α* (TNF-*α*), of which both have bone-resorbing activity in OCPs [104, 105].

#### 3.2.5. ncRNA Targets Involved in the Regulation of Both NF-*κ*B and NFATc1

Phosphoinositide 3-kinase (PI3K)/protein kinase B (AKT) signaling, which is induced by M-CSF binding to CSF1R, could regulate NF-*κ*B activity by elevating phosphorylation and subsequent degradation of I*κ*B [106]. This signaling pathway could also modulate NFATc1 activity by enhancing the formation of inactive form of GSK3-*β* [107]. Upregulation of miR-214, miR-363-3p, and miR-142-5p accelerates PI3K/AKT signaling by targeting phosphatase and tensin homolog (PTEN), which is an inhibitor of PI3K [108–111]. In addition, the excessive expression of lncRNA-TUG1 also gives rise to downregulated PTEN, which may be relevant to the positive effect of TUG1 on osteoclastogenesis [112]. lncRNA-CRNDE also promotes osteoclast proliferation via the PI3K/AKT signaling pathway. Overexpression of CRNDE in osteoclasts significantly decreases the protein levels of p-PI3K, p-AKT, and B cell lymphoma 2 (Bcl-2) [113]. AKT also regulates the transcriptional activity of the forkhead box O (FoxO) family, which counteracts reactive oxygen species (ROS) generation and plays roles in several miRNA pathways in osteoclastogenesis [114]. For example, FoxO3 mediates miR-182 promoting TNF-*α*-induced osteoclastogenesis, and FoxO1 is involved in miR-142-5p promoting osteoclastogenesis [111, 115].

Heme oxygenase (HO-1) deficiency increases the differentiation, survival, and function of osteoclasts by enhancing I*κ*B phosphorylation, promoting the production and nuclear translocation of NFATc2, and elevating levels of intracellular calcium and ROS [116]. miR-183 is a member of the miR-182-183 miRNA cluster and has highly homologous 5′-seed sequences with miR-182. Upregulation of miR-183 positively regulates osteoclastogenesis by attenuating the expression of HO-1 at the posttranscriptional level [117, 118]. Downregulation of miR-1225 potentiates osteoclastogenesis by targeting Keap1 to regulate the Kelch-like ECH-associated protein 1- (Keap1-) nuclear factor erythroid 2-related factor 2- (Nrf2-) HO-1 axis, in which Keap1 overexpression decreases the expression of its downstream proteins, Nrf2 and HO-1 [119].

Transforming growth factor-*β*-induced factor 2 (Tgif2) promotes osteoclastogenesis via a positive feedback loop, as NFATc1, c-Jun, and c-Fos activate Tgif2 transcription and Tgif2 in turn elevates the activity of NFATc1, NF-*κ*B, and c-Jun. Downregulation of miR-34a potentiates osteoclastogenesis by targeting Tgif2, while the overexpression of miR-34a suppresses osteoclastogenesis and bone resorption [120].

#### 3.2.6. Other ncRNA Targets Involved in the Early Differentiation of OCPs

NFATc1, cooperating with AP-1, PU.1, and microphthalmia-associated transcription factor (MITF), generates an osteoclast-specific transcriptional complex and induces the expression of various osteoclast-specific genes, including TRAP, CALCR, CTSK, and osteoclast-associated receptor (OSCAR) [79]. MITF is also thought to be important for the survival of OCPs as it is capable of regulating the antiapoptotic protein Bcl-2 expression in the osteoclast lineage [30]. Downregulation of miR-155, miR-340, miR-141, miR-219, and miR-133a promotes osteoclastogenesis by targeting MITF, and the overexpression of these miRNAs results in decreased expression of osteoclast marker genes and repressed osteoclast differentiation and bone-resorbing activity [31, 55, 121], although the positive effect of miR-133a on osteoclastogenesis is also reported [92]. LPS-induced miR-29b elevates osteoclast survival through targeting and decreasing Bcl-2-modifying factor (BMF), which can bind to Bcl-2 and induce the apoptosis of osteoclasts [122].

V-maf musculoaponeurotic fibrosarcoma oncogene homolog B (MAFB) inhibits osteoclastogenesis by attenuating gene induction of NFATc1 and OSCAR, as well as DNA binding of NFATc1, c-Fos, and MITF, while MAFB also retains the phagocytic activity of BMMs [123]. Upregulation of miR-148a, miR-199a-5p, and miR-338-3p facilitates osteoclastogenesis by targeting MAFB, demonstrated by the increased number of TRAP-positive multinucleated osteoclasts and upregulated expression of osteoclast marker genes [44, 78, 124, 125]. Here, miR-338-3p exhibits contradictory effects on osteoclastogenesis as we mentioned above that it inhibits osteoclastogenesis by targeting IKK*β* and RANKL [62].

Protein inhibitor of activated STAT3 (PIAS3) regulates the transcriptional activity of MITF, NFATc1, and c-Fos, resulting in suppressed expression of NFATc1 and OSCAR [126]. Excessive expression of miR-9718 promotes M-CSF- and RANKL-induced osteoclast differentiation by targeting PIAS3, while miR-9718 inhibition exerts the opposite effects [127].

Sirtuin 1 (SIRT1) suppresses osteoclast differentiation by inhibiting ROS production as well as transient receptor potential cation channel subfamily V member 1 (TRPV1) activation. Upregulation of miR-506 supports osteoclastogenesis by targeting at 3′-UTR of SIRT1, while a miR-506 inhibitor suppresses osteoclastogenesis demonstrated by TRAP staining, impaired functions, reduced the multinuclear cell number, and downregulated the expression of osteoclast markers and correlated genes [128].

The miR-29 family, including miR-29a, miR-29b, and miR-29c, is also involved in osteoclast lineage commitment as it negatively targets G protein-coupled receptor 85 (GPR85) and CD93, which are promoters of monocyte differentiation into macrophages, and thus interferes with osteoclastogenesis indirectly [129].

### 3.3. Involvement of ncRNAs in Mononuclear Osteoclast Fusion

After the early differentiation, OCPs are committed to the osteoclast lineage and expressing various osteoclast-specific genes, including TRAP. These cells are also known as TRAP-positive mononuclear osteoclasts. These TRAP-positive mononuclear osteoclasts then migrate to each other, make contacts with neighboring cells by membrane protrusion, and subsequently fuse to become multinucleated osteoclasts [130].

#### 3.3.1. DC-STAMP and Related Molecules

Dendritic cell-specific transmembrane protein (DC-STAMP), a downstream gene of NFATc1, is essential for the cell-cell fusion of osteoclasts [30, 131]. Downregulation of miR-7b and miR-30a boosts the fusion of TRAP-positive mononuclear osteoclasts by targeting DC-STAMP and modulating the expression of their downstream fusogenic and regulatory genes [132, 133]. Inhibition of DC-STAMP by miR-7b also modulates the expression of NFATc1 and OSCAR via the immunoreceptor tyrosine-based activation motif- (ITAM-) immunoreceptor tyrosine-based inhibition motif (ITIM) network [132]. miR-30a overexpression in OCPs suppresses the DC-STAMP/c-Fos/NFATc1 signaling pathway and thus inhibits the formation of osteoclasts, actin ring, and resorbing pit [133]. The overexpression of miR-26a has the same effect as miR-30a, as it targets and downregulates connective tissue growth factor (CTGF), which induces DC-STAMP expression [134, 135]. lncRNA-MAYA is involved in the regulation of DC-STAMP indirectly as it mediates the activation of yes-associated protein 1 (YAP1), which upregulates CTGF expression, leading to elevated cancer cell-induced osteoclastogenesis and bone resorption [136]. miR-222-3p is also reported to modulate the expression of DC-STAMP [137].

#### 3.3.2. Rho GTPases

The rho family of GTPases, including Ras homolog gene family member A (RhoA), Ras-related C3 botulinum toxin substrate 1 (Rac1), and cell division control protein 42 (CDC42), is involved in various osteoclastogenesis events, such as osteoclast commitment, proliferation, migration, fusion, and maturation [138]. Upregulation of miR-31 could control cytoskeleton organization and support mononuclear osteoclast fusion by targeting RhoA and negatively regulating RhoA expression at an appropriate level as both overexpression and suppression of RhoA show a negative effect on osteoclastogenesis [139]. miR-124 may inhibit the expression of RhoA and Rac1, leading to reduced mononuclear osteoclast migration [80]. Upregulation of miR-29 is implicated in cytoskeletal organization-involved cell migration by targeting CDC42 and SLIT-ROBO Rho GTPase-activating protein 2 (SRGAP2), a negative regulator of Rac1 [129]. Downregulation of miR-17 is reported to increase the fusion of osteoclasts possibly via the miR-17-protein-tyrosine phosphatase- (PTP-) EphA4 axis or directly targeting vav guanine nucleotide exchange factor 3 (Vav3), thus promoting ITGB3-dependent Vav3-mediated activation of Rac1/Rac2 [140].

#### 3.3.3. PKC*α*

Protein kinase C *α* (PKC*α*) is involved in cell-to-cell contact, clustering, and fusion as it mediates rearrangement of microtubule and actin networks. It is also observed that PKC*α* supports cell viability as it regulates antiapoptotic factor Bcl-2. miR-142-3p functions as an inducer of cell death in osteoclasts by targeting PKC*α*. And overexpression of miR-142-3p gives rise to decreased size, viability, and average number of nuclei per cell of osteoclasts [141].

#### 3.3.4. CD226

CD226 is involved in the process of mononuclear osteoclast fusion into multinucleated osteoclasts. It is reported that the formation of multinucleated osteoclasts is impaired when treated with the extracellular domain of CD226, while the formation of TRAP-positive mononuclear osteoclasts is not affected. The promoter of CD226 is regulated by AP-1, which mediates the inhibition effect of NFATc1 on CD226 promoter activity [142]. CD226 is one of the potential targets of miR-422a, and its expression was found to be negatively correlated, albeit not significantly, with that of miR-422a [47].

### 3.4. Involvement of ncRNAs in Multinucleated Osteoclast Function and Survival

Osteoclasts are polarized when initiating bone resorption. Polarized osteoclasts form three distinct membrane domains: a ruffled border, a sealing zone, and a functional secretory domain. At the sealing zone, the actin cytoskeleton forms an attachment ring and anchors osteoclasts to the bone matrix [143, 144]. The ruffled border inside the sealing zone consists of distinctive villous-like complexes of the plasma membrane, which harbor massive “spike-like” vacuolar proton pumps [143]. Via these proton pumps, the ruffled membrane transports protons into the resorption compartment to dissolve minerals, while osteoclasts secrete the lytic enzymes TRAP and pro-CTSK to degrade bone matrix proteins [5]. This erodes the underlying bone. The degradation products of collagen and other matrix components are processed in osteoclasts and transported through the cell and exocytosed through the functional secretory domain [144].

#### 3.4.1. CTSK and CALCR

CTSK and CALCR are activated by a transcriptional complex containing NFATc1 and its cooperators including AP-1, p38MAPK, PU.1, and MITF [30, 145]. CTSK is able to degrade type I collagen and regulate osteoclast actin ring formation and therefore influence bone resorption activity [146]. CALCR mediates the antiapoptotic effect of calcitonin on mature osteoclasts, leading to increased osteoclast survival while decreasing their resorption activity [129]. Downregulation of miR-141 and miR-190 improves osteoclastogenesis by targeting CALCR [55]. Upregulation of miR-29 increases osteoclast-mediated bone resorption by targeting CALCR [129]. However, it is also reported that miR-29b expression decreases during osteoclast differentiation *in vitro*, and its overexpression impairs TRAP expression, lacunae generation, and collagen degradation, which are indicators of osteoclast activity [147].

#### 3.4.2. NFI-A

Reduced nuclear factor I/A (NFI-A) expression is essential for the terminal differentiation of osteoclasts, as well as granulocytes and monocytes. Upregulation of miR-29 positively regulates osteoclastogenesis by targeting NFI-A [129]. NFI-A is also targeted by miR-223 [64]. The role of miR-223 in osteoclastogenesis is still controversial as both of positive and negative effects are mentioned [27, 64, 148, 149]. The expression of miR-223, which is typically downregulated during osteoclastogenesis, is regulated by the competitive binding of NFI-A and CCAAT-enhancer-binding proteins *α* (C/EBP*α*) to their promoters. NFI-A maintains a low level of miR-223 expression, whereas C/EBP*α* upregulates miR-223 expression [150]. miR-223 is a component of the PU.1/miR-223/NFI-A/CSF1R positive feedback loop. In this loop, PU.1 expression is induced by M-CSF-stimulated production of pre-miR-223. And PU.1, together with C/EBP*α*, stimulates the expression of miR-223 reversely. miR-223 then downregulates NFI-A, leading to the upregulation of CSF1R, which actuates osteoclastogenesis, as well as the expression of PU.1, MITF, and other transcription factors [23, 25, 64], while in the condition that miR-223 expression is extremely low, its translational repression on NFI-A will be relieved, resulting in blocked osteoclastogenesis [64].

#### 3.4.3. Cbl and c-Src

Osteoclast adhesion and migration involve the tyrosine phosphorylation of casitas B lineage lymphoma (Cbl) and PI3K, as well as the activation of c-Src. Besides its role as an adaptor protein, Cbl is also a ubiquitin ligase which could target CSF1R and c-Src and regulate the activation of Rac [151, 152]. Downregulated miR-9 and miR-181a promote Cbl expression and activity by targeting Cbl, which is also a potential target of miR-422a [47, 153]. Downregulation of miR-222-3p supports osteoclast adhesion and migration by targeting c-Src, while pit formation activity of multinucleated osteoclasts is decreased by miR-222-3p gain-of-function [137]. CREB-binding protein (CBP, also known as phosphoprotein membrane anchor with glycosphingolipid microdomains 1 (PAG1)), which is also a potential target of miR-422a, inhibits c-Src activity in a dose-dependent manner. Moreover, introduction of CBP leads to suppressed formation of both actin rings and the ruffled border, resulting in decreased bone-resorbing activity [47, 154]. Downregulation of miR-17 elevates c-Src activation by targeting PTP, which dephosphorylates the inhibitory pY527 of c-Src to activate c-Src signaling. miR-20 and miR-92, which are also members of the miR-17~92 cluster genes, also have potential target sites on PTP mRNA, indicating the possibility that they play similar negative regulation roles in osteoclastogenesis to that of miR-17 [140, 155].

#### 3.4.4. APC

miR-27a obviously suppresses osteoclast differentiation and activity as miR-27a overexpression markedly disrupts F-actin ring structure and decreases the pit formation of osteoclasts. Adenomatous polyposis coli (APC) is one of the targets of miR-27a, by which miR-27a enhances the inhibitory effect of estrogen on osteoclastogenesis and bone-resorbing activity [84]. APC can stabilize microtubules directly by interacting with microtubules via its microtubule-binding regions or indirectly by interacting with end-binding protein 1 (EB1). Inhibited APC activity gives rise to decreased microtubule stability in osteoclasts, leading to reduced sealing zone formation as well as decreased bone resorption activity. Phosphorylation by GSK-3*β* decreases the microtubule-binding ability of APC, while AKT enhances APC activity by phosphorylating GSK-3*β* and decreasing its activity [156].

#### 3.4.5. MMP9

Matrix metalloproteinase 9 (MMP9), which is activated by RANKL stimulation, could promote OCP migration and accelerate osteoclastic resorption via increased degradation of collagen types I and IV [157]. Downregulation of miR-218 augments osteoclastogenesis by negatively targeting MMP9 [158]. MMP13 is also involved in osteoclast differentiation and activation as it is reported to activate pre-MMP9 and complement the activity of MMP9 in dissolving the bone matrix [159]. miR-126-5p targets MMP13 and represses its expression at a posttranscriptional level to regulate osteoclast differentiation and bone-resorbing activity in a giant cell tumor [160].

#### 3.4.6. MMP14

MMP14 is crucial for osteoclast migration. As a type of secreted MMP, MMP14 is able to regulate osteoclast podosome function, and its collagenolytic activity is thought to be helpful for the osteoclast movement through a collagen fiber network. MMP14-deficient osteoclasts exhibit decreased bone resorption activity [161]. miR-133a targets MMP14 and thus represses osteoclast differentiation and bone-resorbing activity [55].

#### 3.4.7. Fas and FasL

The Fas-Fas ligand (FasL) system, which provides an important apoptotic mechanism for osteoclasts, is activated by FasL binding to a Fas receptor [67, 162]. miR-21 is involved in the inhibiting and proapoptotic effects of estrogen during osteoclastogenesis as estrogen downregulates miR-21 biogenesis. Decreased miR-21 expression increases the protein levels of FasL, a target of miR-21, and thus induces osteoclastic apoptosis [163]. miR-21 also participates in the regulation of osteoclast differentiation and recruitment in aging bones. Decreased miR-21 levels in aging bones result in osteocyte apoptosis and thus induce osteoclastogenesis by high-mobility group box-1- (HMGB1-) receptor for advanced glycation end product (RAGE) signaling [164]. HMGB1-RAGE signaling plays important roles in regulating actin cytoskeleton reorganization in osteoclasts, thereby participating in RANKL-induced osteoclastogenesis [165]. So miR-21 is also likely to be involved in the regulation of osteoclast bone-resorbing activity.

#### 3.4.8. Rab27a

Rab27a is capable of regulating the transport of lysosome-related organelles and cell surface receptors and thus participating in the modulation of osteoclast bone-resorbing activity. Additionally, Rab27a-deficient osteoclasts display multinucleation, larger cell appearance, and abnormal actin ring formation [166]. miR-124 directly targets Rab27a. Overexpression of miR-124 reduces the protein level of Rab27a, thus inhibiting osteoclastogenesis and impairing the functions of osteoclasts [167].

#### 3.4.9. Other ncRNAs and Their Targets Involved in Osteoclast Function and Survival

Interferon regulatory factor 1 (IRF1), one of the targets of miR-132, suppresses osteoclast maturation and/or activity under the mechanism related to proinflammatory cytokines like IL-12 and IL-18 [33, 168].

Thrombospondin 1 (THBS1), a glycoprotein present in a variety of cell types, is one of the targets of miR-let-7e. THBS1 has been identified to be involved in osteoclast-mediated resorption under an undefined mechanism [33, 169].

miR-16-5p suppresses RANKL-induced osteoclastogenesis in a giant cell tumor of bone and disrupts the formation of the F-actin ring, which is essential for osteoclast bone-resorbing activity. However, the direct target of miR-16-5p functioning is undefined [170].

### 3.5. Involvement of ncRNAs with Undefined Targets and Functioning Stages

#### 3.5.1. lncRNA-Bmncr

The expression of lncRNA-Bmncr gradually decreases during RANKL-induced osteoclastogenesis, which reaches the lowest level at 72 h. The overexpression of Bmncr reduces osteoclast numbers and bone-resorbing capacity and decreases the expression of osteoclast-specific genes, while knockdown of Bmncr has opposite effects [171].

#### 3.5.2. miR-146a-5p

Compared with psoriasis patients without arthritis and normal controls, miR-146a-5p expression is higher in peripheral CD14^+^ monocytes from patients with psoriatic arthritis. miR-146-5p induces osteoclast activation and bone-resorbing activity as shown by miR-146a-5p knockdown revealing a decreased generated osteoclast number and impaired bone resorption [172].

#### 3.5.3. Enhancer RNAs

Enhancer RNAs (eRNAs) are a kind of lncRNA present in the 5′-flanking regions or intronic regions of genes which initiate bidirectional RNA biosynthesis and are involved in the differentiation of various cell types [173, 174]. Deletion of the eRNA regions of neuropilin 2, DC-STAMP, and NFATc1 significantly inhibits the formation of TRAP-positive multinucleated cells, indicating that their expression positively regulates osteoclastogenesis. And eRNAs also functions by increasing the expression of RNAs encoding osteoclast-related proteins [173].

## 4. Discussion

Bone remodeling involves various biological events of osteoblasts and osteoclasts, all of which are regulated by a precise network of systemic and local factors, including ncRNAs [175, 176]. When the network is unable to compensate for the difference between bone resorption and synthesis, unbalanced bone remodeling will cause diseases [177]. As a kind of ncRNAs with numerous numbers, miRNAs downregulate gene expression by either of two posttranscriptional ways: mRNA cleavage or translational repression. The former one occurs when the mRNA has sufficient complementarity to the miRNA; otherwise, the latter one occurs [178]. The way lncRNAs work is mainly through the interaction with miRNAs as molecular sponges [179].

Most studies of the functions of ncRNAs in the regulation of osteoclast activity have focused on ncRNA expression levels, the effects of ncRNA mimics/inhibitors, and ncRNA target genes. Apart from osteoclast pathological experiments, RAW264.7 cells and BMMs have been the most frequently used sources of osteoclasts in such studies. RAW264.7 is a mouse monocyte macrophage leukemia cell line developed by injection of Abelson leukemia virus [180]. BMMs are produced by culturing nonadherent bone marrow cells from the femur and tibia in the presence of M-CSF [181]. The J774 and U-937 cell lines have also been used to generate osteoclasts [180]. Reverse transcription quantitative polymerase chain reaction (RT-qPCR) analysis is one of the most frequently used methods for analyzing the gene expression levels of ncRNAs and osteoclast-specific genes [58, 97, 101, 180, 182, 183]. Microarray analysis is also widely used for detecting gene expression levels, as well as identifying novel regulators of osteoclastogenesis [141, 184]. Microcomputed tomography enables the assessment of the structural parameters involved, such as bone volume per total volume (BV/TV), mean trabecular number (Tb.N), mean trabecular thickness (Tb.Th), and trabecular separation (Tb.Sp) [43, 180, 185]. Immunoblotting, as well as qualitative and semiquantitative analyses, is extensively used for the detection and characterization of proteins [186]. Chromatin immunoprecipitation (ChIP) enables the analysis of interactions between proteins and DNA in chromosomal fragments and the identification of the binding sites of transcription factors and other proteins [180, 187]. Additionally, four web-based miRNA databases, miRBase (http://www.mirbase.org), TargetScan (http://www.targetscan.org), PicTar (http://www.pictar.mdc-berlin.de), and miRanda (http://www.microRNA.org), are essential for bioinformatics analysis [108]. Assays of pit formation, actin ring formation, cell migration, macrophage commitment, and apoptosis are also widely used to evaluate the functions and generation of osteoclasts [86, 98, 115, 129, 147, 153].

## 5. Conclusion

With the profound research in bone physiology and diseases related to bone metabolism disorder, the essential role of ncRNAs in osteoclastogenesis is gradually recognized. In this review, we summarized the current knowledge of ncRNAs involved in important osteoclast differentiation events, including OCP generation, early differentiation, mononuclear osteoclast fusion, and multinucleated osteoclast function and survival. Nevertheless, there are still some unknown mechanisms for ncRNAs regulating osteoclast differentiation, and further work is needed to supplement and perfect the effects of ncRNAs, particularly those of lncRNAs, circRNAs, and eRNAs, on the functions of osteoclasts.

## Figures and Tables

**Table 1:** Major ncRNAs related to biological events in osteoclasts.

miRNA (miR)	Validated target(s)	Functions	Expression	Effect(s)	References
lncRNA-LINC00311	DLL3	Positive	Not mentioned	Promotes osteoclast proliferation and differentiation, shifts cell cycle distribution, and inhibits apoptosis	[101]
lncRNA-AK077216	Not mentioned	Positive	Upregulated	Inhibits OCP apoptosis and promotes osteoclast differentiation, fusion, bone-resorbing activity, and actin ring formation	[98]
lncRNA-CRNDE	Not mentioned	Positive	Not mentioned	Promotes osteoclast proliferation and shifts cell cycle distribution	[113]
lncRNA-MAYA	Not mentioned	Positive	Not mentioned	Promotes osteoclast differentiation and bone-resorbing activity	[136]
lncRNA-MIRG	Not mentioned	Positive	Upregulated	MIRG inhibition decreases osteoclast differentiation and bone-resorbing activity	[83]
lncRNA-MALAT1	Not mentioned	Positive	Not mentioned	EPC-derived exosomal MALAT1 promotes osteoclast differentiation and migration	[82]
lncRNA-DANCR	Not mentioned	Positive	Not mentioned	Knockdown of DANCR reduces the promotion effect of compression force on osteoclastogenesis	[104, 105]
lncRNA-TUG1	Not mentioned	Positive	Not mentioned	Promotes osteoclast proliferation and inhibits osteoclast apoptosis	[112]
lncRNA-Jak3	Not mentioned	Positive	Upregulated	Regulates the Jak3-NFATc1-CTSK axis; lncRNA-Jak3 knockdown inhibits osteoclast differentiation and bone-resorbing activity	[100]
circRNA_28313	Not mentioned	Positive	Upregulated	circRNA_28313 knockdown inhibits osteoclastogenesis and actin ring formation	[29]
miR-21	PDCD4, FasL	Positive	Upregulated	Downregulates PDCD4 and forms a c-Fos/miR-21/PDCD4 positive feedback loop and promotes osteoclast differentiation and bone-resorbing activity while inhibiting apoptosis	[23, 26, 78, 117, 163, 164, 185, 188–191]
miR-29	NFI-A, CALCR, CDC42, SRGAP2, GPR85, CD93	Positive	Upregulated	Promotes osteoclast differentiation, survival, and bone-resorbing activity	[78, 117, 129, 190, 191]
miR-214	PTEN	Positive	Upregulated	Downregulates PTEN and regulates the PTEN-PI3K-AKT axis, promotes osteoclast activity, and mediates osteoclast-osteoblast intercellular crosstalk	[108, 109, 117, 190, 192]
miR-182	PKR	Positive	Upregulated	Downregulates PKR, promotes osteoclast differentiation and bone-resorbing activity	[88, 115]
miR-183	HO-1	Positive	Upregulated	Downregulates HO-1, promotes osteoclastogenesis	[117, 118, 190]
miR-31	RhoA	Positive	Upregulated	Negatively regulates RhoA expression at an appropriate level; miR-31 inhibition inhibits osteoclast formation, bone-resorbing activity, and actin ring formation	[78, 117, 139, 191]
miR-34c	LGR4	Positive	Upregulated	Downregulates LGR4, promotes the formation of actin rings and TRAP-positive multinucleated cells	[97]
miR-148a	MAFB	Positive	Upregulated	Downregulates MAFB, promotes osteoclastogenesis	[78, 117, 123, 124, 190]
miR-9718	PIAS3	Positive	Unregulated	Downregulates PIAS3, promotes osteoclastogenesis	[117, 126, 127, 190]
miR-483-5p	IGF2	Positive	Not mentioned	Downregulates IGF2, promotes osteoclast differentiation and survival	[71]
miR-506	SIRT1	Positive	Upregulated	Downregulates SIRT1; a miR-506 inhibitor suppresses osteoclastogenesis	[128]
miR-363-3p	PTEN	Positive	Upregulated	Targets PTEN to activate the PI3K/AKT signaling pathway, promotes osteoclastogenesis	[110]
miR-125a-5p	TNFRSF1B	Positive	Upregulated	Downregulates TNFRSF1B, promotes osteoclastogenesis, OCP proliferation, and growth, migration, and invasion of RAW264.7 cells	[66]
miR-142-5p	PTEN	Positive	Upregulated	Targets PTEN to activate the PI3K/AKT signaling pathway, promotes osteoclast differentiation and activity	[111]
miR-199a-5p	MAFB	Positive	Upregulated	Suppresses MAFB, promotes osteoclast differentiation	[125]
miR-145-5p	OPG	Positive	Not mentioned	Suppresses OPG, promotes osteoclastogenesis	[49]
miR-212	IL-15, CX3CR1	Positive	Upregulated	Downregulates IL-15; miR-212 inhibition impairs osteoclastogenesis	[33]
miR-132	IRF1	Positive	Upregulated	Downregulates IRF1; miR-132 inhibition impairs osteoclastogenesis	[33]
miR-99b	IGF1R	Positive	Upregulated	Downregulates IGF1R; miR-99b inhibition impairs osteoclastogenesis	[33]
miR-let-7e	TNFAIP3, ITGA4, THBS1	Positive	Upregulated	Downregulates TNFAIP3, ITGA4, and THBS1; miR-let-7e inhibition impairs osteoclastogenesis	[33]
miR-146a-5p	Not mentioned	Positive	Not mentioned	miR-146a-5p knockdown impairs osteoclast differentiation and bone resorption	[172]
lncRNA-NONMMUT037835.2	Not mentioned	Negative	Downregulated	Inhibits osteoclastogenesis	[40]
lncRNA-Bmncr	Not mentioned	Negative	Downregulated	Inhibits osteoclast differentiation and bone-resorbing activity	[171]
miR-218	TNFR1, TLR4, MMP9	Negative	Downregulated	Inhibits the p38MAPK-c-Fos-NFATc1 pathway, represses TNFR1, TLR4, and MMP9, inhibits osteoclastogenesis, cell migration, actin ring formation, and bone resorption	[58, 73, 86, 117, 158, 190]
miR-34a	Tgif2	Negative	Downregulated	Downregulates Tgif2, inhibits osteoclast differentiation and bone-resorbing activity	[117, 120, 190, 191]
miR-155	MITF, Socs1	Negative	Downregulated	Downregulates MITF, PU.1, and Socs1, inhibits osteoclast fusion, differentiation, and bone-resorbing activity	[23, 31, 78, 89, 90, 190, 191]
miR-7b	DC-STAMP	Negative	Downregulated	Downregulates DC-STAMP, inhibits osteoclast differentiation and bone-resorbing activity	[117, 132]
miR-26a	CTGF	Negative	Upregulated	Downregulates CTGF; inhibits osteoclastogenesis, actin ring formation, and bone-resorbing activity	[117, 134, 190]
miR-124	NFATc1, Rab27a, IL-11	Negative	Downregulated	Downregulates NFATc1, Rab27a, and IL-11, inhibits the proliferation and motility of OCPs, and decreases osteoclast differentiation and activity	[37, 78, 80, 117, 166, 167, 190, 191]
miR-125a	TRAF6, TNFAIP3	Negative/positive	Downregulated/upregulated	Inhibited by NFATc1; inhibits osteoclast differentiation and downregulates TRAF6; miR-125a inhibition suppresses osteoclastogenesis and upregulates TNFAIP3	[33, 57, 78, 117, 190, 191]
miR-146a	TRAF6	Negative	Downregulated/upregulated	Downregulates TRAF6, inhibits osteoclastogenesis	[56, 77, 78, 117, 190, 191, 193, 194]
miR-503	RANK	Negative	Not mentioned	Downregulates RANK, inhibits osteoclast differentiation and bone-resorbing activity	[38, 78, 117, 190, 195]
miR-145	Smad3	Negative	Downregulated	Downregulates Smad3, inhibits osteoclast differentiation and bone-resorbing activity	[60]
miR-142-3p	PKC*α*	Negative	Downregulated	Downregulates PKC*α*, inhibits osteoclast clustering, fusion, differentiation, and survival	[141]
miR-222-3p	Potential: c-Src	Negative	Downregulated	Negatively regulates c-Src, inhibits osteoclast differentiation and bone-resorbing activity	[137]
miR-340	MITF	Negative	Downregulated	Downregulates MITF, inhibits osteoclastogenesis	[121, 185]
miR-16-5p	Not mentioned	Negative	Downregulated	Inhibits osteoclastogenesis and disrupts the structure of F-actin rings	[170]
miR-126-5p	MMP13	Negative	Not mentioned	Downregulates MMP13, inhibits osteoclast differentiation and bone-resorbing activity	[159, 160]
miR-17	PTP, Vav3 (potential)	Negative	Not mentioned	miR17/PTP/EphA4 regulatory axis: decreased miR-17 upregulates PTP, PTP downregulates EphA4, which activates osteoclastogenesis; may downregulate Vav3 and thus inhibits osteoclast fusion	[140, 155, 196]
miR-141	MITF, CALCR, EphA2	Negative	Downregulated	Downregulates MITF, CALCR, and EphA2; inhibits osteoclast differentiation and activity	[52, 55]
miR-219	MITF, TRAF6	Negative	Downregulated	Downregulates MITF and TRAF6, inhibits osteoclast differentiation and activity	[55]
miR-190	CALCR	Negative	Downregulated	Downregulates CALCR, inhibits osteoclast differentiation and activity	[55]
miR-23a	GSK3-*β*	Negative	Downregulated	Inhibits GSK3-*β* activation, inhibits osteoclast differentiation and activity	[95]
miR-1225	Keap1	Negative	Downregulated	Downregulates Keap1, inhibits osteoclast differentiation and activity	[119]
miR-100-5p	FGF21	Negative	Downregulated	Downregulates FGF21, inhibits osteoclastogenesis	[50]
miR-27a	PPAR*γ*, APC	Negative	Not mentioned	Downregulates PPAR*γ* and APC, inhibits osteoclast differentiation and activity in physiologic condition and ovariectomized (OVX) mice	[84]
miR-618	TLR4	Negative	Downregulated	Inhibits osteoclastogenesis via the TLR4/MyD88/NF-*κ*B signaling pathway	[73]
miR-30a	DC-STAMP	Negative	Downregulated	Downregulates DC-STAMP, inhibits osteoclast formation, differentiation, and activity	[133]
miR-144-3p	RANK	Negative	Downregulated	Downregulates RANK, inhibits osteoclast differentiation, formation, proliferation, and apoptosis	[39]
miR-9	Cbl	Negative	Not mentioned	Downregulates Cbl, inhibits osteoclast survival and OCP migration	[153]
miR-181a	Cbl	Negative	Not mentioned	Downregulates Cbl, inhibits osteoclast survival and OCP migration	[153]
miR-29b	BMF	Negative/positive	Upregulated/downregulated	Downregulates BMF; knockdown impairs osteoclast migration, commitment, and formation; inhibits osteoclast differentiation and activity; enhances osteoclast survival	[122, 129, 147]
miR-378	Not mentioned	Negative/positive	Upregulated	Overexpression and underexpression of miR-378 inhibit osteoclastogenesis	[55, 76–78]
miR-223	NFI-A, IKK*α* (potential)	Negative/positive	Downregulated	Promotes osteoclastogenesis by regulating the relative expression of NFI-A and CSF1R, downregulates IKK*α*, and inhibits osteoclastogenesis	[25, 27, 64, 77, 78, 117, 148, 150, 190]
miR-133a	MITF, MMP14, CXCL11, CXCR3, SLC39A1	Negative/positive	Upregulated	Downregulates MITF, MMP14, CXCL11, CXCR3, and SLC39A1; both positive and negative effects on osteoclastogenesis are reported	[55, 75, 78, 92, 117, 197]
miR-106b	RANKL	Negative/positive	Not mentioned	Downregulates RANKL, has different regulatory roles in CIA bone microenvironment and giant cell tumor of bone	[41, 43]
miR-422a	Potential: Cbl, CD226, IGF1, CBP, TOB2	Positive	Upregulated	Unclear (probably inhibits those five inhibitors of osteoclastogenesis)	[47, 78, 117]
miR-338-3p	IKK*β*, MAFB, RANKL	Negative/positive	Upregulated/downregulated	Downregulates IKK*β*, MAFB, and RANKL; both positive and negative effects on osteoclastogenesis are reported	[42, 44, 62]

**Table 2:** ncRNAs implicated in biological events in osteoclasts in *in vivo* studies.

ncRNA	Sample resource(s)	Functions	Effect(s)	References
LINC00311	OVX mice injected with LINC00311 intraperitoneally	Positive	Accelerates osteoclast growth, increases osteoclast numbers, promotes osteoclast proliferation, and inhibits osteoclast apoptosis	[101]
MAYA	MAYA-knockdown mice (by shRNA or locked nucleic acid)	Positive	Reduces bone lesions and breast cancer bone tumor burden	[136]
MALAT1	Femur fracture mice treated with exosomes derived from EPCs transfected with MALAT1-targeting siRNA	Positive	Inhibits osteoclastogenesis and bone healing	[82]
circRNA_28313	circRNA_28313-knockdown OVX mice (Lsh-circRNA_28313-infected)	Positive	Exhibits increased BV/TV, Tb.N, Tb.Th, and Tb.Sp	[29]
miR-21	miR-21-knockout mice	Positive	Exhibits inhibited bone resorption, osteoclast differentiation and activity, and increased bone mass	[189, 198]
miR-214	Osteoclastic miR-214 transgenic mice	Positive	Exhibits increased osteoclast activity and bone resorption and reduced bone mineral density (BMD)	[109, 192, 199]
mir-182	(1) mir-182^flox/flox^LysMcre(+) mice (2) mir-182^+/+^LysMcre(+) mice	Positive	Compared with (2), (1) exhibits increased bone mass, connectivity density, BV, BMD, and Tb.N and decreased trabecular bone spacing	[88]
miR-148a	Silencing of miR-148a with an antagomir	Positive	Inhibits bone resorption and increases bone mass	[124]
miR-9718	Silencing of miR-9718 with an antagomir in OVX mice and sham-operated control mice	Positive	Exhibits inhibited bone resorption and increased bone mass in both OVX mice and sham-operated control mice	[127]
miR-145-5p	miR-145-5p agomir-treated collagen-induced arthritis (CIA) mice	Positive	Enhances bone erosion	[49]
miR-34a	(1) miR-34a-knockout and heterozygous mice (2) Osteoclastic miR-34a transgenic mice	Negative	(1) Exhibits increased bone resorption and reduced bone mass (2) Exhibits inhibited bone resorption and increased bone mass	[120]
miR-155	miR-155^−/−^ CIA mice	Negative	Exhibits reduced generation of osteoclasts and local bone destruction	[200]
miR-124	(1) Adjuvant-induced arthritis mice treated with pre-miR-124 (2) Nude mice transplanted with MDA-MB-231 cells (breast cancer cell line) expressing miR-124	Negative	(1) Exhibits reduced osteoclast numbers and activity and inhibited osteoclast differentiation (2) Inhibits bone metastasis of breast cancer cells and cancer cell-induced osteolysis and shows relieved bone lesion	[37, 201]
miR-146a	CIA mice injected with miR-146a	Negative	Less osteoclasts at the border of the pannus and bone	[56]
miR-503	(1) Silencing of miR-503 with and antagomir (2) Overexpression of miR-503 with agomir	Negative	(1) Increases RANK protein expression and bone resorption and reduces bone mass (2) Inhibits bone resorption and bone loss	[38]
miR-145	Bilateral OVX-operated mice treated with a miR-145 agomir	Negative	Attenuates trabecular disconnection and separation and reverses the increase in the RANKL to OPG ratio	[60]
miR-17~92	miR-17~92 osteoclast conditional knockout mice	Negative	Exhibits decreased connectivity density, BV/TV, BMD, Tb.N, and Tb.Th and increased trabecular separation and bone resorption	[155]
miR-141	(1) Mice injected with pre-miR-141 (2) Nude mice inoculated with SCP28 cells via intracardiac injection and then injected with pre-miR-141 (3) Aged rhesus monkeys with miR-141 delivery into osteoclasts via a nucleic acid delivery system	Negative	((1) and (2)) Exhibits decreased bone lesions, osteoclast numbers and activity, and breast cancer bone tumor burden (3) Exhibits decreased bone erosion and osteoclast numbers and activity	[52, 55]
miR-219	(1) Mice injected with pre-miR-219 (2) Nude mice inoculated with SCP28 cells via intracardiac injection and then injected with pre-miR-219	Negative	((1) and (2)) Exhibits decreased bone lesions, osteoclast numbers and activity, and breast cancer bone tumor burden	[55]
miR-190	Mice injected with pre-miR-190	Negative	Reduces osteoclast activity	[55]
miR-100-5p	OVX mice treated with agomir-miR-100-5p	Negative	Decreases osteoclast numbers and the expression of osteoclast-specific genes	[50]
miR-27a	OVX mice injected with miR-27a-carrying chitosan	Negative	Inhibits osteoclast differentiation and activity	[84]
miR-223	CIA mice injected with lentiviral vectors expressing a miR-223 target sequence	Negative/positive	Exhibits decreased osteoclastogenesis, osteoclast numbers and bone erosion, and relatively intact bone	[202]
miR-133a	Spinal tuberculosis rabbit injected with notochordal cells containing miR-133a (1) mimic or (2) inhibitor (3) Mice intravenously injected with pre-miRNA-133a (4) miR-133a knockdown in OVX rats by injecting with antagomiR-133a	Negative/positive	(1) Exhibits reduced osteoclast numbers (2) Opposite to (1) (3) Reduces osteoclast activity (4) Decreases bone erosion and levels of osteoclastogenesis-related factors in serum and increases lumbar spine BMD	[55, 92, 197]
miR-106b	CIA mice injected with (1) miR-106b mimics or (2) inhibitor OVX mice injected with (3) agomiR-106b or (4) antagomiR-106b	((1) and (2)) Positive ((3) and (4)) Negative	(1) Similar to NC (2) Suppresses bone destruction and reduces osteoclast numbers and the RANKL/OPG ratio (3) Increases BMD and other bone parameters and decreases osteoclast formation and activity (4) Opposite to (3)	[41, 43]

## Abbreviations

Akt: Protein kinase B
AP-1: Activator protein 1
APC: Adenomatous polyposis coli
Bcl-2: B cell lymphoma 2
BMD: Bone mineral density
BMF: Bcl-2-modifying factor
BMMs: Bone marrow-derived macrophages
BV/TV: Bone volume per total volume
C/EBP*α*: CCAAT-enhancer-binding proteins *α*
CALCR: Calcitonin receptor
Cbl: Casitas B lineage lymphoma
CBP: CREB-binding protein
CDC42: Cell division control protein 42
ChIP: Chromatin immunoprecipitation
CIA: Collagen-induced arthritis
circRNAs: Circular RNAs
CMPs: Common myeloid progenitors
CSF1R: Colony-stimulating factor 1 receptor
CTGF: Connective tissue growth factor
CTSK: Cathepsin K
CX3CR1: CX3C chemokine receptor 1
CXCL11: Chemokine (C-X-C motif) ligand 11
CXCR3: Chemokine (C-X-C motif) receptor 3
DC-STAMP: Dendritic cell-specific transmembrane protein
DLL3: Delta-like 3
EB1: End-binding protein 1
EPC: Endothelial progenitor cells
EphA: Ephrin type-A receptor
ERK: Extracellular signal-regulated kinase
eRNA: Enhancer RNA
FasL: Fas ligand
FGF21: Fibroblast growth factor 21
Foxo: Forkhead box O
GMPs: Granulocyte/macrophage progenitors
GPR85: G protein-coupled receptor 85
GSK3-*β*: Glycogen synthase kinase 3-*β*
HER3: Human epidermal growth factor receptor 3
HMGB1: High-mobility group box-1
HO-1: Heme oxygenase
HSCs: Hematopoietic stem cells
IFN: Interferon
IGF1: Insulin-like growth factor 1
I*κ*B: Inhibitor of nuclear factor *κ*B
IKK: Inhibitor of nuclear factor *κ*B kinase subunit
IRF1: Interferon regulatory factor 1
ITAM: Immunoreceptor tyrosine-based activation motif
ITGA4: Integrin subunit *α*4
ITGB3: Integrin *β*3
ITIM: Immunoreceptor tyrosine-based inhibition motif
Jak3: Janus kinase 3
Keap1: Kelch-like ECH-associated protein 1
LGR4: Leucine-rich repeat-containing G protein-coupled receptor 4
lincRNAs: Long or large intergenic ncRNAs
lncRNAs: Long noncoding RNAs
MAFB: V-maf musculoaponeurotic fibrosarcoma oncogene homolog B
Maml1: Mastermind-like protein 1
MAPK: Mitogen-activated protein kinase
M-CSF: Macrophage colony-stimulating factor
miRNAs: MicroRNAs
MITF: Microphthalmia-associated transcription factor
MMP: Matrix metalloproteinase
MSU: Monosodium urate monohydrate
MyD88: Myeloid differentiation primary response gene 88
ncRNAs: Noncoding RNAs
NFATc1: Nuclear factor of activated T cell cytoplasmic 1
NFI-A: Nuclear factor I/A
NF-*κ*B: Nuclear factor *κ*-light-chain-enhancer of activated B cells
NIP45: NFAT-interacting protein
Notch2: Neurogenic locus notch homolog protein 2
Nrf2: Nuclear factor erythroid 2-related factor 2
OCPs: Osteoclast precursors
OPG: Osteoprotegerin
OSCAR: Osteoclast-associated receptor
OVX: Ovariectomized
PAG1: Phosphoprotein membrane anchor with glycosphingolipid microdomains 1
PDCD4: Programmed cell death 4
PI3K: Phosphoinositide 3-kinase
PIAS3: Protein inhibitor of activated STAT3
piRNAs: Piwi-interacting RNAs
PKC*α*: Protein kinase C *α*
PKR: Protein kinase double-stranded RNA-dependent
PPAR*γ*: Peroxisome proliferator-activated receptor *γ*
PTEN: Phosphatase and tensin homolog
PTP: Protein-tyrosine phosphatase
Rac: Ras-related C3 botulinum toxin substrate
RAGE: Receptor for advanced glycation end products
RANK: Receptor activator of nuclear factor *κ*B
RANKL: Receptor activator of nuclear factor *κ*B ligand
RhoA: Ras homolog gene family member A
RISC: RNA-induced silencing complex
ROR1: Receptor tyrosine kinase-like orphan receptor 1
ROS: Reactive oxygen species
RT-qPCR: Reverse transcription quantitative polymerase chain reaction
SCF: Stem cell factor
siRNAs: Small interfering RNAs
SIRT1: Sirtuin 1
Smad3: Mothers against decapentaplegic homolog 3
snoRNAs: Small nucleolar RNAs
Socs1: Suppressor of cytokine signaling 1
SRGAP2: SLIT-ROBO rho GTPase-activating protein 2
STAT3: Signal transducer and activator of transcription-3
TAB1: Transforming growth factor-*β*-activated kinase 1-binding protein 1
TAK1: Transforming growth factor-*β*-activated kinase 1
Tb.N: Trabecular number
Tb.Sp: Trabecular separation
Tb.Th: Trabecular thickness
Tgif2: Transforming growth factor-*β*-induced factor 2
THBS1: Thrombospondin 1
TLR4: Toll-like receptor 4
TNF-*α*: Tumor necrosis factor-*α*
TNFAIP3: Tumor necrosis factor-*α*-induced protein 3
TNFR: Tumor necrosis factor receptor
TNFRSF1B: Tumor necrosis factor receptor superfamily member 1B
TOB2: Transducer of ERBB2, 2
TRAF6: Tumor necrosis factor receptor-associated factor 6
TRAP: Tartrate-resistant acid phosphatase
TRPV1: Transient receptor potential cation channel subfamily V member 1
Vav3: Vav guanine nucleotide exchange factor 3
YAP1: Yes-associated protein 1.
